# Expression and regulation of neurotrophic and angiogenic factors during human intervertebral disc degeneration

**DOI:** 10.1186/s13075-014-0416-1

**Published:** 2014-08-20

**Authors:** Abbie LA Binch, Ashley A Cole, Lee M Breakwell, Anthony LR Michael, Neil Chiverton, Alison K Cross, Christine L Le Maitre

**Affiliations:** Biomedical Research Centre, Sheffield Hallam University, Howard Street, Sheffield, S1 1WB UK; Sheffield Teaching Hospitals, Sheffield, UK

## Abstract

**Introduction:**

The degenerate intervertebral disc (IVD) becomes innervated by sensory nerve fibres, and vascularised by blood vessels. This study aimed to identify neurotrophins, neuropeptides and angiogenic factors within native IVD tissue and to further investigate whether pro-inflammatory cytokines are involved in the regulation of expression levels within nucleus pulposus (NP) cells, nerve and endothelial cells.

**Methods:**

Quantitative real-time PCR (qRT-PCR) was performed on 53 human IVDs from 52 individuals to investigate native gene expression of neurotrophic factors and their receptors, neuropeptides and angiogenic factors. The regulation of these factors by cytokines was investigated in NP cells in alginate culture, and nerve and endothelial cells in monolayer using RT-PCR and substance P (SP) protein expression in interleukin-1 (IL-1β) stimulated NP cells.

**Results:**

Initial investigation on uncultured NP cells identified expression of all neurotrophins by native NP cells, whilst the nerve growth factor (NGF) receptor was only identified in severely degenerate and infiltrated discs, and brain derived neurotrophic factor (BDNF) receptor expressed by more degenerate discs. BDNF expression was significantly increased in infiltrated and degenerate samples. SP and vascular endothelial growth factor (VEGF) were higher in infiltrated samples. *In vitro* stimulation by IL-1β induced NGF in NP cells. Neurotropin-3 was induced by tumour necrosis factor alpha in human dermal microvascular endothelial cells (HDMECs). SP gene and protein expression was increased in NP cells by IL-1β. Calcitonin gene related peptide was increased in SH-SY5Y cells upon cytokine stimulation. VEGF was induced by IL-1β and interleukin-6 in NP cells, whilst pleiotrophin was decreased by IL-1β. VEGF and pleiotrophin were expressed by SH-SY5Y cells, and VEGF by HDMECs, but were not modulated by cytokines.

**Conclusions:**

The release of cytokines, in particular IL-1β during IVD degeneration, induced significant increases in NGF and VEGF which could promote neuronal and vascular ingrowth. SP which is released into the matrix could potentially up regulate the production of matrix degrading enzymes and also sensitise nerves, resulting in nociceptive transmission and chronic low back pain. This suggests that IL-1β is a key regulatory cytokine, involved in the up regulation of factors involved in innervation and vascularisation of tissues.

## Introduction

The intervertebral disc (IVD) is considered the largest aneural structure within the human body and is composed of three main anatomical regions; the central nucleus pulposus (NP), constrained by the annulus fibrosus (AF) and the cartilaginous end plate. During degeneration, nociceptive nerves are seen to accompany blood vessels into the IVD [1-3], yet the mechanisms of how this occurs are unclear. Chronic low back pain is widespread, affecting 80% of the population at some point in their lives, with 40% of cases attributed to degeneration of the IVD [4], costing the economy millions each year [5]. The link between IVD degeneration and pain production was traditionally thought to be due to compression of the nerve root, but links have been shown between neural ingrowth and painful discs [6], leading to new ideologies emerging [7,8]. IVD degeneration is characterised by the loss of extracellular matrix and altered biomechanical properties caused by an imbalance between anabolic and catabolic factors [9,10]. Cytokines such as interleukin-1 (IL-1β) and tumour necrosis factor alpha (TNFα) are known for their role within IVD degeneration and herniation [11-18] and lead to the upregulation of matrix-degrading enzymes, mainly matrix metalloproteinases MMP-3 and MMP-13 and a disintegrin and metalloproteinase with thrombospondin motifs ADAMTS-1, ADAMTS-4 and DAMTS-5 [18-24], which cleave both aggrecan and collagen type II. Infiltration of blood vessels and nerve fibres is considered a characteristic feature of IVD degeneration [25] and is associated with increased pain sensation.

Current literature surrounding the mechanisms of low back pain associated with neural ingrowth have concentrated on factors that promote ingrowth and survival of nerves, such as the neurotrophic factors, nerve growth factor (NGF), brain-derived neurotrophic factor (BDNF) and neurotrophin-3 (NT3), along with their receptors, the tropomyosin kinases TrkA, TrkB and TrkC [26-31]. Freemont and colleagues was the first group to identify the nonmyelinated nerve fibres in IVDs associated with pain, which were also shown to express Substance P (SP) [1]. The same group later identified that these nerve fibres expressed TrkA and accompanied microvessels into the degenerate IVD that expressed NGF [6]. Abe and colleagues found that human NP cells constitutively express NGF and BDNF, yet expression increases in response to IL-1β and TNFα [32]. TNFα also induced SP mRNA expression from IVD cells [30], implicating a role in neoinnervation of the degenerate IVD. Nerve ingrowth is often seen to follow the track of ingrowing blood vessels, with neurotrophic factors expressed by blood vessels proposed to drive the neuronal ingrowth [6]. Angiogenic factors include pleiotrophin and vascular endothelial cell growth factor (VEGF), which have been shown to be produced by human IVD cells, with increased expression seen in discs with higher numbers of blood vessels [33,34]. However, whether the expression of these factors alters during degeneration has not been investigated to date, and limited studies have investigated their regulation by cytokines.

This study aimed to identify a wide range of neurotrophic factors along with their receptors, pain-related peptides and angiogenic factors at the mRNA level from RNA directly extracted from degenerate human NP tissue, and to further investigate whether proinflammatory cytokines are involved in the regulation of their expression in human NP, nerve and endothelial cells.

## Materials and methods

### Human tissue

Human IVD tissue was obtained from surgery or postmortem examination with informed consent of the patient or relatives. Ethical approval was obtained from Sheffield Research Ethics Committee (09/H1308/70). Details of all patient samples are presented in Table 1. IVD tissue was obtained from 51 patients undergoing microdiscectomy for nerve root compression, cauda equine syndrome or sciatica and from two postmortem samples from one individual.

**Table 1:** **Patient details of NP cells used within this study**

**Code**	**Source**	**Age**	**IVD level**	**IVD intact**	**Average grade**	**Infiltrated**
1	Surgical	32	L5/S1	Yes	2.0	
2	Postmortem	45	L4/L5	Yes	2.0	
3	Postmortem	45	L3/L4	Yes	3.0	
4	Surgical	51	L4/L5		2.0	
5	Surgical	42	L5/S1	Yes	3.0	
6	Surgical	24	L4/L5	No	3.0	
7	Surgical	38	L5/S1	No	3.5	
8	Surgical	25	L4/L5	Yes	4.0	
9	Surgical	45	L5/S1	Yes	4.0	
10	Surgical	43	L5/S1	No	4.0	
11	Surgical	47	L5/S1	Yes	5.3	
12	Surgical	47	L5/S1	Yes	5.85	
13	Surgical	26	C3/C5		6.0	
14	Surgical	30	L5/S1	No	6.15	
15	Surgical	46	L5/S1	No	6.16	
16	Surgical	70	C5/C6		6.18	
17	Surgical	65	L3/L4	No	6.5	
18	Surgical	52	L4/L5	No	6.7	
19	Surgical	66	L5/S1	Yes	6.85	
20	Surgical	43	L5/S1	No	7.0	
21	Surgical	44	L5/S1	Yes	7.0	
22	Surgical	38	L5/S1		7.0	
23	Surgical	36	L5/S1	Yes	8.0	
24	Surgical	28	L5/S1	No	8.0	
25	Surgical	35	L5/S1	Yes	8.0	
26	Surgical	33	L4/L5	Yes	8.0	
27	Surgical	37	L5/S1	Yes	8.0	
28	Surgical	49	L4/L5	No	8.0	
29	Surgical	29	L5/S1	No	8.0	
30	Surgical	42	L4/L5	No	8.0	
31	Surgical	38	L4/L5	No	8.15	
32	Surgical	42	L5/S1	No	9.0	
33	Surgical	38	C6/C7	No	9.0	
34	Surgical	48	L4/L5	No	10.0	
35	Surgical	26	L5/S1	No	11.0	
36	Surgical	35	L4/L5	No	11.0	
37	Surgical	49	L2/L3	No	1.0	Yes
38	Surgical	20	L4/L5	No	3.0	Yes
39	Surgical	40	L5/S1	No	3.0	Yes
40	Surgical	39	L4/L5	No	3.0	Yes
41	Surgical	42	L4/L5	No	4.0	Yes
42	Surgical	26	L4/L5	Yes	4.0	Yes
43	Surgical	31	L5/S1	No	4.0	Yes
44	Surgical	39	L5/S1	No	5.0	Yes
45	Surgical	62	L3/L4	Yes	6.0	Yes
46	Surgical	40	L5/S1	Yes	7.0	Yes
47	Surgical	33	L5/S1	Yes	9.0	Yes
**48**	**Surgical**	**45**	**L4/L5**	**No**	**3.0**	
**49**	**Surgical**	**27**	**L5/S1**	**No**	**3.0**	
**50**	**Surgical**	**21**	**L5/S1**	**No**	**7.0**	**Yes**
**51**	**Surgical**	**38**	**L4/L5**	**No**	**7.0**	**Yes**
**52**	**Surgical**		**L5/S1**	**No**	**8.0**	
**53**	**Surgical**	**28**	**L4/L5**	**Yes**	**9.0**	

Eight patient samples were classified as nondegenerate (grade 0 to 3), 12 patient samples were classed as moderately degenerate (grade 4 to 6), 20 IVD samples were classified as severely degenerate (grade 7 to 12) and 12 patients had evidence of infiltration. Note, samples 2 and 3 are from the same postmortem individual. Patients in bold were used in cytokine treatment experiments. Samples 48, 50, 51, 52 and 53 were used for interleukin-1β treatments. Samples 49, 51 and 52 were used in interleukin-6 and tumour necrosis factor alpha treatments. NP, nucleus pulposus; IVD, intervertebral disc.

### Tissue processing

Tissue consisting of AF and NP was fixed in 10% neutral buffered formalin, and processed to paraffin wax. Following embedding, 4 μm tissue sections were taken for haematoxylin and eosin staining, and stained sections were evaluated independently by two researchers (ALAB and CLLM) to determine the extent of degenerative tissue changes. Sections were scored numerically between 0 and 12 based on the presence of cell clusters, fissures, loss of demarcation and haematoxophilia (indicating reduced proteoglycan content); a score of 0 to 3 indicates a histologically normal (nondegenerate) IVD and a grade ≥4 indicates evidence of degeneration, as described previously [18,35]. Interobserver scores were averaged and assigned to each tissue sample. Sections were also taken for routine immunohistochemical analysis of CD11b expression. CD11b is a leukocyte adhesion molecule and was used to identify immune cell infiltrates in NP tissue samples from prolapsed IVDs. CD11b immunohistochemical analysis was performed as described previously [35]. Samples were classified as infiltrated on the basis of CD11b immunopositivity. On account of the heterogeneity often observed in prolapsed IVD tissues [36], serial sections were made from paraffin-embedded tissues that had matched cDNA samples derived from surgically obtained NP. Routine histological and immunohistochemical examinations were made on multiple sections, at different levels throughout the paraffin-embedded tissue, to ensure assessment of any heterogeneity. cDNA samples were only considered to be from nondegenerate tissue if all haematoxylin and eosin-stained sections were assigned a histological grade of degeneration <4. cDNA samples were considered to be from an infiltrated IVD if CD11b immunopositivity (indicating leukocyte presence) was observed in any tissue section evaluated.

### Native nucleus pulposus cell extraction and investigations of native neurotrophic and angiogenic factor expression

#### Isolation of disc cells

Samples from degenerate IVD tissue were obtained from 51 patients undergoing microdiscectomy for the treatment of low back pain and from two postmortem samples from one individual. NP tissue was separated from contaminating AF and end plate and digested with 2 U/ml protease (Sigma, Poole, UK) in Dulbecco’s modified Eagle’s media for 30 minutes at 37°C, and washed twice with Dulbecco’s modified Eagle’s media. Following this, NP cells were isolated using 2 mg/ml collagenase type 1 (Sigma Aldrich) for 4 hours at 37°C. Cells were passed through a 40 μm cell strainer (Sigma Aldrich, Paisley, UK) as published previously [37]. Following extraction, cells were used for either direct RNA extraction or cell culture.

#### Quantitative real-time polymerase chain reaction on directly extracted RNA from human nucleus pulposus cells

Quantitative real-time polymerase chain reaction (qRT-PCR) was conducted on nine nondegenerate samples (grade 0 to 3) from eight patients, 12 moderately degenerate samples (grade 4 to <7) from 12 patients, and 19 severely degenerate samples (grade 7 to 12) from 19 patients, and 13 samples with evident infiltration from patients experiencing pain (Table 1). Extracted RNA was subjected to treatment with DNase (Qiagen, Manchester, UK) and purified using the Qiagen MinElute Cleanup kit prior to cDNA synthesis using Bioscript RT enzyme (Bioline, London, UK) and random hexamers (Applied Biosystems, Paisley, UK) [37].

qRT-PCR was performed on a StepOnePlus™ Real-Time PCR System (Applied Biosystems) in order to investigate gene expression levels of neurotrophic factors NGF, BDNF and NT3 and their receptors TrkA, TrkB and TrkC, along with pain-related peptides SP and calcitonin gene-related peptide (CGRP) and angiogenic factors VEGF and pleiotrophin (Applied Biosystems) within directly extracted samples from degenerate and nondegenerate samples. Glyceraldehyde-3-phosphate dehydrogenase and 18S (Applied Biosystems) were used as housekeeping genes to allow normalisation. Ten-microlitre reactions were prepared using the TaqMan Universal PCR Master Mix (Applied Biosystems). Results were analysed using the 2^–ΔCt^ method and presented as relative gene expression normalised to the average cycle threshold for the two housekeeping genes.

### Cytokine regulation of neurotrophic factors, angiogenic factors and pain peptides in human nucleus pulposus cells, SH-SY5Y cells and human dermal microvascular endothelial cells

#### Monolayer nucleus pulposus cell culture

Following extraction, NP cells were plated out into culture flasks for expansion in monolayers, and were cultured in Dulbecco’s modified Eagle’s media supplemented with 10% v/v heat-inactivated foetal calf serum, 100 U/ml penicillin, 100 μg/ml streptomycin, 250 ng/ml amphotericin, 2 mM glutamine (Invitrogen) and 50 μg/ml ascorbic acid (Sigma Aldrich) (Complete cell culture media) and maintained at 37°C in a humidified atmosphere containing 5% carbon dioxide. Complete culture media was changed every 3 days.

#### Alginate culture

To redifferentiate NP cells towards their native phenotype, cells were cultured in alginate beads that have been shown previously to maintain NP phenotype [18]. Alginate culture also enables RNA extraction and gene expression analysis because cells can be released easily from three-dimensional culture for analysis. Following expansion in monolayers up to passage 2, NP cells were suspended in 1.2% medium viscosity sodium alginate (Sigma Aldrich) at 4 × 10^6^ cells/ml and alginate beads formed via dropping through a 19-gauge needle into 200 mM CaCl_2_ (Sigma Aldrich). After incubation at 37 °C for 10 minutes, beads were washed twice with 0.15 M NaCl (Sigma Aldrich) before being washed twice in complete media. All NP cells used in this experiment were cultured in alginate for 2 weeks prior to cytokine treatment to enable the cells to regain native cellular phenotypes [38].

#### Monolayer SH-SY5Y and human dermal microvascular endothelial cell culture

SH-SY5Y neuroblastoma cells were differentiated by a 7-day pretreatment with 5 μM retinoic acid prior to seeding into a six-well plate at a cell density of 5 × 10^5^ cells/ml. Human dermal microvascular endothelial cells (HDMEC) were seeded into six-well plates at a density of 1 × 10^6^ cells/ml in complete HDMEC growth factor media (PromoCell, Heidelberg, Germany, UK).

### Cytokine treatments

#### Human nucleus pulposus cell cytokine treatments

Following 2-week culture in alginate beads, NP cells were treated with proinflammatory cytokines known to be upregulated during IVD degeneration and herniation, including IL-1β, IL-6 and TNFα (Peprotech, London UK) for 48 hours. NP cells were treated with IL-6 and TNFα at 0, 1, 10 and 100 ng/ml in serum-free media in triplicate for three independent patients (Table 1). IL-1β treatments were performed for five patients (Table 1) in triplicate (0, 1, 10, 100 ng/ml) and for three patients (Table 1) in triplicate at a wider concentration range (0, 0.001, 0.01, 0.1, 1, 10 and 100 ng/ml) for 48 hours to identify dose responses. Conditioned media were collected after 48 hours and used for further analysis.

#### SH-SY5Y and HDMEC cytokine treatments

SH-SY5Y and HDMEC cells were allowed to adhere for 24 hours prior to stimulation with 10 ng/ml IL-1β, IL-6, IL-8 or TNFα for 48 hours.

#### RNA extraction and cDNA synthesis

Following cytokine treatments, triplicate alginate beads were used for the analysis of a number of neurotrophic factors and their receptors, pain-related peptides and angiogenic factors. RNA was extracted using TRIzol reagent (Gibco, Paisley, UK). Prior to TRIzol extraction, alginate beads were washed in 0.15 M NaCl and dissolved by application of alginate dissolving buffer (55 mM sodium citrate (Na_3_C_6_H_5_O_7_), 30 mM Na ethylenediamine tetraacetic acid and 0.15 M NaCl at pH 6.8) for 15 minutes at 37°C followed by digestion of extracellular matrix in 0.06% w/v collagenase type 1 (Sigma) for 30 minutes.

Extracted RNA was subjected to treatment with DNase (Qiagen) and purified using the Qiagen MinElute Cleanup kit prior to cDNA synthesis using Bioscript RT enzyme (Bioline) and random hexamers (Applied Biosystems) [37]. Target genes were investigated using qRT-PCR.

#### Real-time polymerase chain reaction

qRT-PCR was performed as described previously to investigate gene expression levels of predesigned assays for neurotrophic factors NGF, BDNF and NT3 and their receptors TrkA, rkB and TrkC, along with pain-related peptides SP and CGRP and angiogenic factors VEGF and pleiotrophin (Applied Biosystems). The qRT-PCR data were analysed using the 2^–ΔΔCt^ method where treatments were compared with untreated controls as published previously [39].

#### Statistical analysis

qRT-PCR expression data were found to be nonparametric in distribution so the Kruskall–Wallis with Conover–Inman *post hoc* analysis test was used to identify significant differences between treatments *(P* ≤ 0.05).

#### Substance P enzyme-linked immunosorbent assay

Human SP peptide was identified in cell culture media from IL-1β-stimulated NP cells. Human NP cells were cultured in alginate beads as described previously and were stimulated with 1, 10 or 100 ng/ml for 48 hours, after which conditioned media was collected and SP levels were analysed using a SP colourimetric enzyme linked immunosorbent assay kit as per the manufacturer’s instructions (ab133029; Abcam, Cambridge, UK). Data analysis was conducted using the Kruskall–Wallis with Conover–Inman *post hoc* analysis to identify significant differences between treatments *(P* ≤ 0.05).

## Results

### Identification of neurotrophic factors and their receptors within native IVD tissue

**Figure 1:**
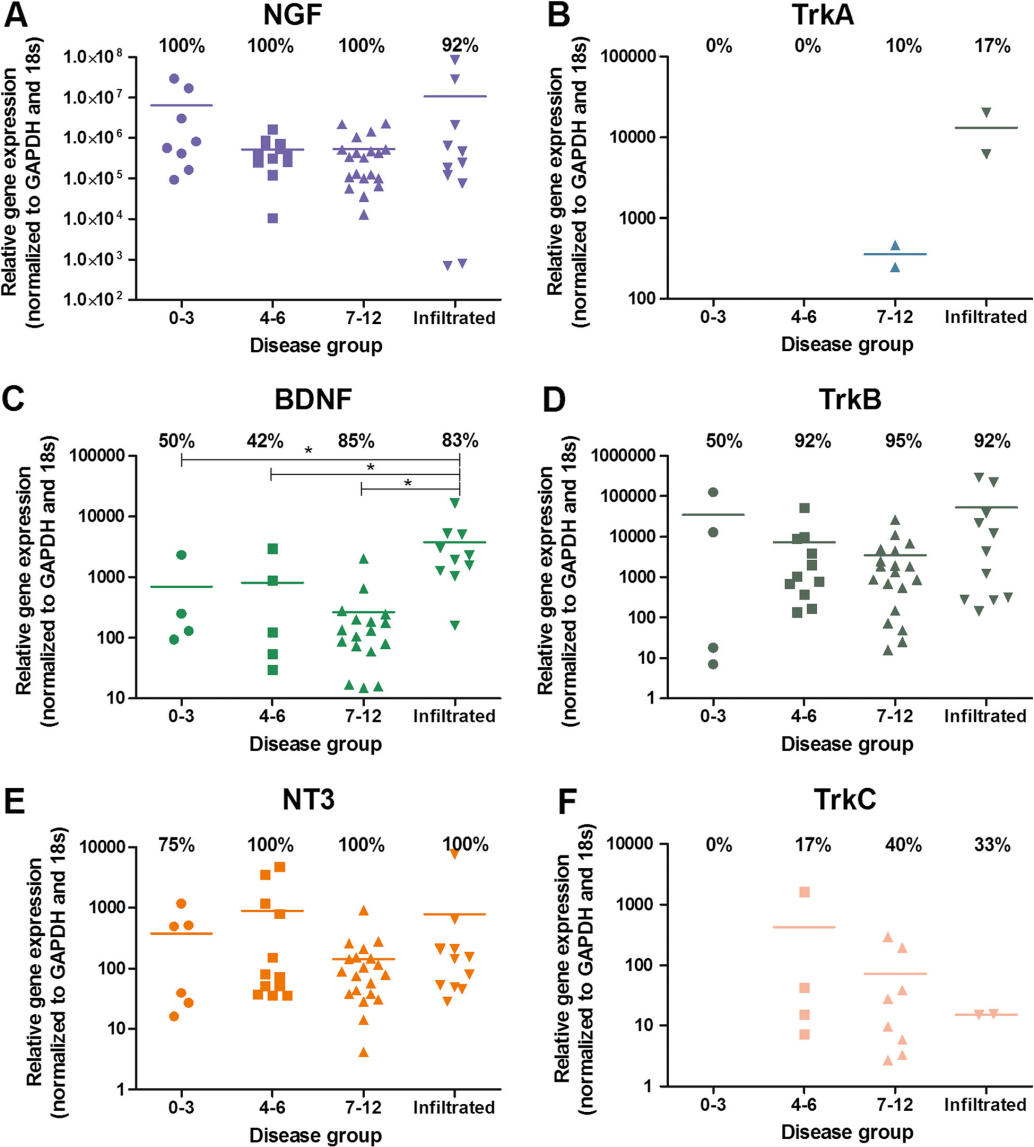
**Identification of neurotrophic factors and their receptors within native intervertebral disc tissue.** Percentage expression is represented as the number of disc samples in each cohort that expressed the target gene, shown by the number above the bars. Bars represent the levels of mRNA expression seen in these samples. Expression of neurotrophic nerve growth factor (NGF) **(A)** and its receptor tropomyosin kinase (Trk)A **(B)**, of brain-derived neurotrophic factor (BDNF) **(C)** and its receptor TrkB **(D)**, and of neurotrophin 3 (NT3) **(E)** and its receptor TrkC **(F)** in human intervertebral discs graded histologically: grade 0 to 3, *n* = 8; grade 4 to 6, *n* = 12; grade 7 to 12, *n* = 20; infiltrated, *n* = 12 samples of intervertebral disc tissue. *Statistical significance between levels of gene expression: *P* ≤ 0.05. GAPDH, glyceraldehyde-3-phosphate dehydrogenase.

Directly extracted NP cells were investigated by qRT-PCR for the native expression of neurotrophic factors and their receptors, all of which were identified at the mRNA level irrespective of their classification. NGF demonstrated constitutive expression and was present in 100% of nondegenerate and degenerate samples, and in 92% of infiltrated samples (Figure 1A). NGF receptor TrkA was expressed in 0% of nondegenerate (grade 0 to 3) samples, yet expression increased to 10% in severely degenerate samples (grade 7 to 12) and to 17% in samples with the presence of infiltrating cells (Figure 1B). Similarly, BDNF receptor TrkB was expressed in 50% of nondegenerate samples, increasing to 92% expression in moderately (grade 4 to 6) and severely degenerate samples and to 92% in infiltrated samples (Figure 1D). BDNF expression was also present in 50% of nondegenerate samples (Figure 1C), with percentage expression increasing to 85% and 83% within the severely degenerate and the infiltrated cohorts respectively. The levels of expression within the infiltrated cohort were significantly higher compared with the nondegenerate group (*P* = 0.0471). Expression levels within the infiltrated cohort were significantly higher than for both the moderately degenerate group (*P* = 0.0058) and the severely degenerate group (*P* < 0.0001). The number of samples expressing BDNF also increased within the severely degenerate and the infiltrated groups, as opposed to the nondegenerate group (Figure 1C). NT3 was expressed in 75% of nondegenerate samples, yet expression increased to 100% within degenerate and infiltrated disease groups (Figure 1E). Conversely, NT3 receptor TrkC was expressed in very few samples in each cohort, with no expression evident within the nondegenerate group, and only 33% and 40% of degenerate disc samples displaying TrkC gene expression (Figure 1F).

### Pain-related peptide expression in native IVD tissue

**Figure 2:**
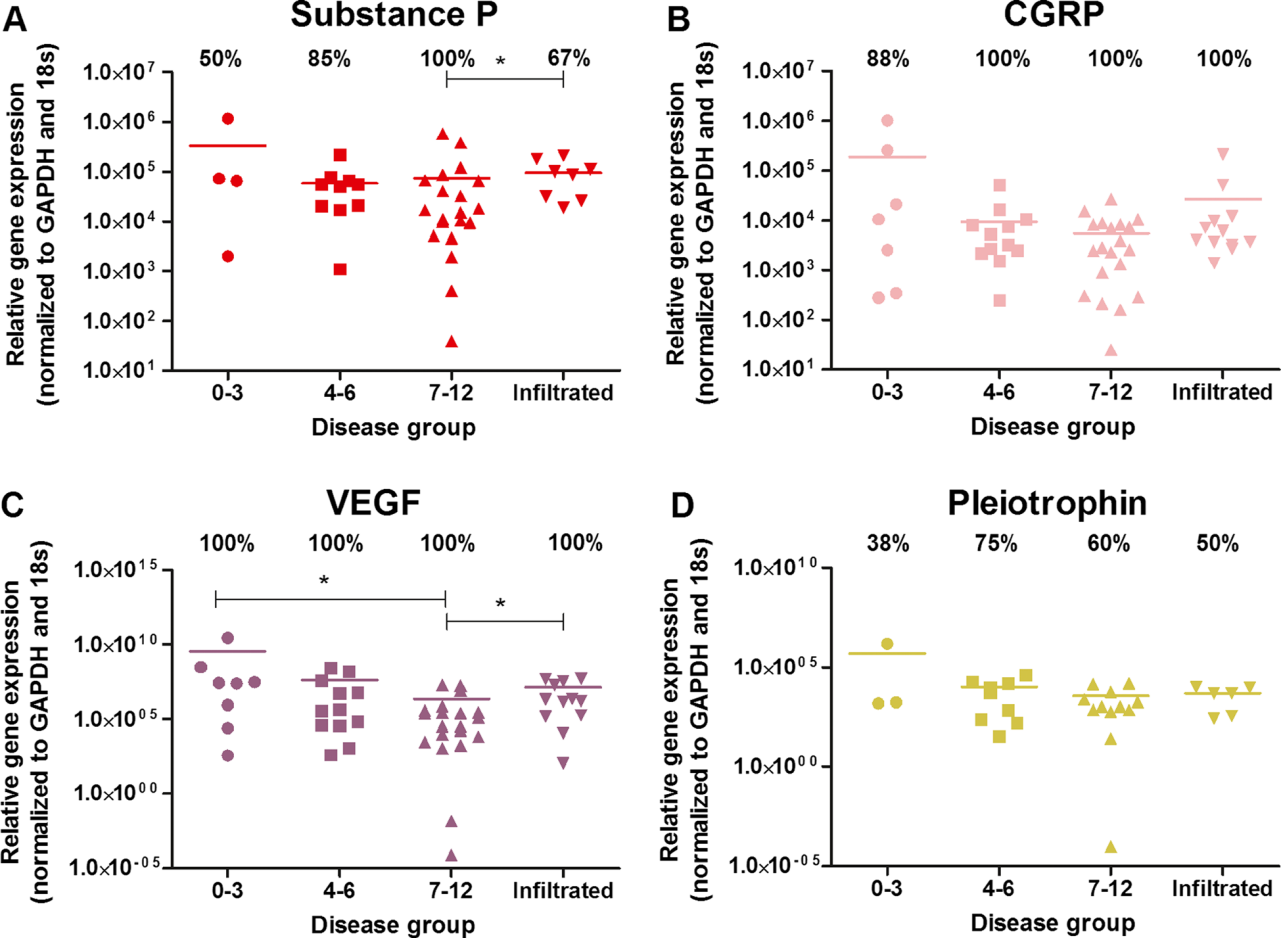
**Neuropeptide and angiogenic factor expression within native intervertebral disc tissue.** Percentage expression is represented as the number of disc samples in each cohort that expressed the target gene, shown by the number above the bars. Bars represent the levels of mRNA expression seen in these samples. Expression of pain-related peptides substance P **(A)** and calcitonin gene-related peptide (CGRP) **(B)** and angiogenic factors vascular endothelial growth factor (VEGF) **(C)** and pleiotrophin **(D)** in human intervertebral discs graded histologically: grade 0 to 3, *n* = 8; grade 4 to 6, *n* = 12; grade 7 to 12, *n* = 20; infiltrated, *n* = 12. GAPDH, glyceraldehyde-3-phosphate dehydrogenase. *Statistical significance: *P* ≤ 0.05.

Native NP cells expressed neuropeptides in all disease groups (Figures 2A and 2B). SP was expressed by 50% of nondegenerate samples, increasing to 100% expression within the severely degenerate group (Figure 2A). Expression levels of SP was significantly higher in the severely degenerate cohort compared with those with evidence of infiltration (*P* = 0.0235). CGRP was present in 88% of nondegenerate samples (Figure 2B) and expression increased to 100% within both the degenerate and infiltrated samples, although the mean level of expression is lower in these groups compared with the nondegenerate samples (Figure 2B).

**Figure 3:**
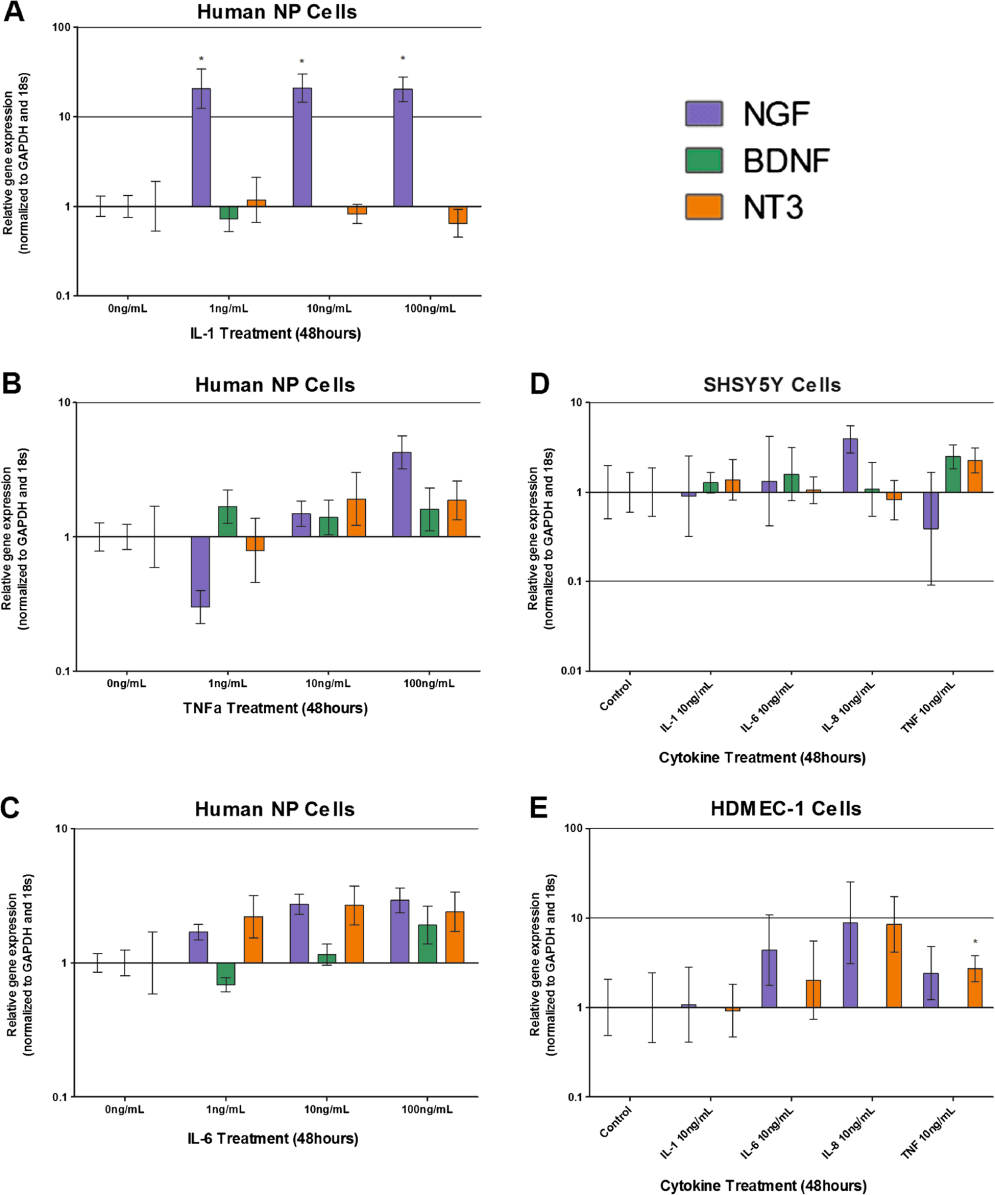
**Cytokine regulation of neurotrophic factors NGF, BDNF and NT3 in human nucleus pulposus cells, SH-SY5Y cells and HDMEC cells.** Neurotrophic factor mRNA expression in human nucleus pulposus (NP) cells stimulated by interleukin (IL)-1 **(A)**, tumour necrosis factor alpha (TNFα) **(B)** and IL-6 **(C)**. Neurotrophic factor mRNA expression in SH-SY5Y neuronal cells following cytokine stimulation **(D)**. Neurotrophic factor mRNA expression in human dermal microvascular endothelial cells (HDMEC; endothelial cell line) following cytokine stimulation **(E)**. *Statistical significance: *P* ≤ 0.05. BDNF, brain-derived neurotrophic factor; GAPDH, glyceraldehyde-3-phosphate dehydrogenase; NGF, nerve growth factor; NT3, neurotrophin 3.

### Angiogenic factors within native IVD tissue

Angiogenic factors VEGF and pleiotrophin were investigated for their presence within native NP tissue at the mRNA level. VEGF was constitutively expressed in 100% of patient samples irrespective of disease classification (Figure 2C). Interestingly, the levels of expression in nondegenerate samples was significantly higher than the severely degenerate cohorts (*P* = 0.0103). Additionally, expression levels of VEGF seen within the severely degenerate group were significantly higher than the level of expression by infiltrated samples (*P* = 0.028). Pleiotrophin (Figure 2D) was expressed by 38% of nondegenerate samples, rising to 75% in moderately degenerate and 60% in severely degenerate samples, yet no significance was reached.

### Neurotrophic factor regulation by cytokines

Following the identification of neurotrophic factor expression in native NP tissue, NP cells derived from degenerate IVDs were cultured and stimulated with proinflammatory cytokines to determine modulation by inflammatory cytokines expressed during IVD degeneration. Following IL-1β treatment for 48 hours, human NP cells significantly up regulated their expression of neurotrophic factor NGF at concentrations ≥0.1 ng/ml (*P* < 0.001) (Figure 3A), suggesting that IL-1β may be involved in the regulation of neurotrophic factor production from NP cells. Treatment with IL-6 and TNFα, however, failed to significantly regulate neurotrophic factor production (Figure 3B,C), although a small nonsignificant increase was seen following 100 ng/ml TNFα (Figure 3B) and 10 and 100 ng/ml IL-6 (Figure 3C). IL-1β treatment did not have an effect on BDNF or NT3 expression (Figure 3A), whilst small nonsignificant increases in BDNF and NT3 were seen following TNFα (Figure 3B) and IL-6 (Figure 3C).

Neurotrophic factor expression within SH-SY5Y cells was not significantly altered following cytokine treatment at 10 ng/ml for 48 hours (Figure 3D). NT3 was significantly upregulated by 10 ng/ml TNFα (*P* ≤ 0.05) stimulation in HDMEC compared with untreated controls (Figure 3E), whereas BDNF was not expressed by HDMEC (Figure 3E). IL-8 stimulation at 10 ng/ml caused an increase in expression of NT3 in HDMEC, yet this failed to reach significance (Figure 3E).

### Neuropeptide regulation by cytokines

**Figure 4:**
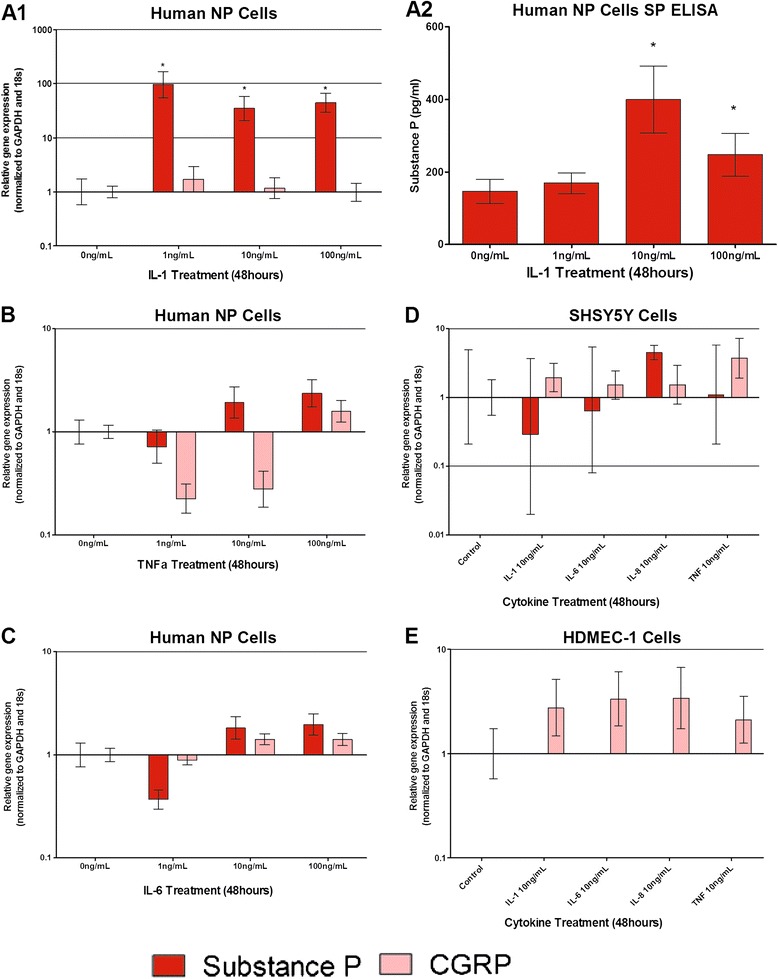
**Cytokine regulation of neuropeptides substance P and CGRP within human nucleus pulposus cells, SH-SY5Y and HDMEC cells.** Substance P (SP) and calcitonin gene-related peptide (CGRP) mRNA expression in human nucleus pulposus (NP) cells following stimulation with interleukin (IL)-1β **(A1)**, tumour necrosis factor alpha (TNFα) **(B)** and IL-6 **(C)**. SP protein was also determined in human NP cells following stimulation with IL-1β **(A2)**. SP and CGRP mRNA expression in SH-SY5Y neuronal cells following cytokine stimulation **(D)**. SP mRNA was not expressed by human dermal microvascular endothelial cells (HDMEC)-1 and cytokines did not significantly regulate CGRP mRNA expression **(E)**. *Statistical significance: *P* ≤ 0.05. GAPDH, glyceraldehyde-3-phosphate dehydrogenase.

Neuropeptide expression following cytokine stimulation was investigated by qRT-PCR. SP expression by human NP cells (Figure 4A1,A2) was significantly increased 100-fold in response to 1 ng/ml IL-1β treatment for 48 hours. Higher doses of IL-1β caused similar effects on SP expression (Figure 4A1). Lower concentrations of IL-1β (1,10 and 100 pg/ml) were tested on three patient samples in triplicate (data not shown) and interestingly even 1 pg/ml IL-1β stimulation for 48 hours resulted in significant upregulation of SP (*P* < 0.0001) compared with controls. Conditioned media collected from IL-1β-treated NP cells demonstrated 400 pg/ml SP protein after 10 ng/ml IL-1β treatment for 48 hours (Figure 4A2), which was significantly increased from 150 pg/ml in untreated NP cells (Figure 4A2). TNFα and IL-6 treatments on human NP cells induced SP expression at doses ≥10 ng/ml (Figure 4B,C) yet this failed to reach significance. CGRP expression was not significantly regulated by cytokine treatments (Figure 4A1,B,C). SP and CGRP expression was not regulated by cytokine stimulation within SH-SY5Y cells (Figure 4D), and SP was not expressed by HDMEC cells (Figure 4E).

### Angiogenic factor regulation by cytokines

**Figure 5:**
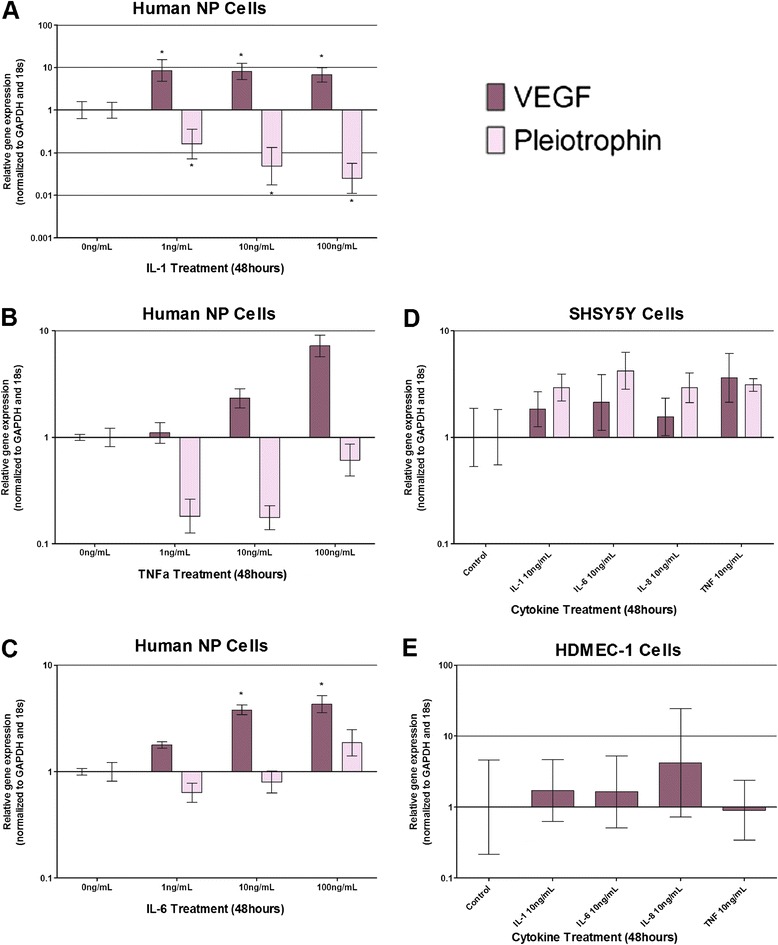
**Cytokine regulation of angiogenic factors VEGF and pleiotrophin in human nucleus pulposus cells, SH-SY5Y and HDMEC cells.** Vascular endothelial growth factor (VEGF) and pleiotrophin mRNA expression in human nucleus pulposus (NP) cells following stimulation with interleukin (IL)-1β **(A)**, tumour necrosis factor alpha (TNFα) **(B)** and IL-6 **(C)**. VEGF and pleiotrophin mRNA expression in human NP cells following cytokine stimulation **(D)**. Human dermal microvascular endothelial cells (HDMEC) cells did not express pleiotrophin mRNA, and cytokines nonsignificantly upregulated VEGF mRNA expression **(E)**. *Statistical significance: *P* ≤ 0.05. GAPDH, glyceraldehyde-3-phosphate dehydrogenase.

VEGF and pleiotrophin were both significantly regulated by IL-1β (Figure 5A). VEGF was upregulated 10-fold (*P* < 0.001) in response to 1 to 10 ng/ml IL-1β treatment. Pleiotrophin, however, was downregulated 50-fold (*P* < 0.001) following 100 ng/ml IL-1β treatment. Pleiotrophin was also downregulated by TNFα (Figure 5B) and IL-6 (Figure 5C) in NP cells. IL-6 treatment at 10 and 100 ng/ml caused a significant increase in VEGF expression in human NP cells, yet pleiotrophin expression was not regulated by IL-6 in NP cells (Figure 5C).

No significant changes in expression levels of VEGF and pleiotrophin were observed in SH-SY5Y and HDMEC when compared with untreated controls (Figure 5D,E).

## Discussion

This study aimed to identify a number of factors involved in innervation and vascularisation of the human IVD during degeneration. Neurotrophins and their receptors, angiogenic factors and neuropeptides were positively identified at the mRNA level within a wide range of NP samples from histologically graded nondegenerate and degenerate IVDs. Further to this, proinflammatory cytokines known to be upregulated within IVD degeneration [12,13,17,18,21,35,40,41], particularly IL-1β, were shown to regulate such factors, leading to the hypothesis that NP cells may be responsible for promoting innervation and vascularisation.

During disc degeneration and herniation, nerve and blood vessels ingrow into deep layers of the IVD, which are thought to contribute to the painful phenotype of the degenerate IVD consequently leading to chronic low back pain [6,42-46]. Early studies identified NGF-expressing blood vessels accompanying sensory nerve fibres into the inner AF and NP [6], which produce neurotransmitters involved in pain transmission within the peripheral and central nervous systems [47]. However, to date limited studies have shown ingrowth into the NP tissue, and those which do demonstrate the track for this is normally via the endplates rather than through the full thickness of the AF. Blood vessels have been shown to track along annular fissures, which could provide deeper access to the NP [48]. In addition, blood vessels have been shown to extensively infiltrate into herniated NP material [33], which is thought at least in part to be involved in the resorption of herniated NP [33]. However, there is an urgent need for further investigation into the presence, localisation and extent of nerve and blood vessels during disc degeneration. This study identified the presence of neurotrophic factors NGF, BDNF and NT3 alongside their receptors TrkA, TrkB and TrkC, pain-related peptides SP and CGRP and angiogenic factors VEGF and pleiotrophin within native IVD tissue, and found their expression to be modulated by cytokines. Krock and colleagues demonstrated recently NGF and BDNF protein production by IVD organ cultures from degenerate disc were significantly higher than those from healthy discs [49]. In the current study, all discs displayed mRNA expression for NGF; however, previous studies have found increased levels of NGF expression in surgical degenerate IVD compared with postmortem tissues [27,50]. The current study only included two postmortem tissues so it was not possible to separate these data, which could explain the lack of correlation seen in the current study. However Purmessur and colleagues also found high levels of NGF and BDNF within IVDs, with no significant changes between regions of the IVD and disease severity [30], which agrees with mRNA expression of NGF shown in the current study. BDNF gene expression in our current study, however, was seen at significantly higher levels in degenerate and infiltrating discs and in more degenerate samples than nondegenerate samples, which confirms the recent results of Krock and colleagues suggesting these neurotrophic factors are increased within degenerate discs [49].

Nerve ingrowth is mostly seen in moderately and severely degenerate discs, and so it may have been presumed that NGF expression would have been increased in these disease groups at the mRNA level. We have shown constitutive expression of NGF within 100% of IVD samples tested, although as the majority of the samples are from diseased tissues this is perhaps not surprising. Richardson and colleagues demonstrated that the neurite outgrowth induced by NP cells derived from degenerate discs was inhibited in the presence of an NGF inhibitor [29], demonstrating the clear role for NP cell-derived NGF in neurite outgrowth. Interestingly, TrkA, the high-affinity receptor for NGF, was only expressed in severely degenerate and infiltrated discs, suggesting that NGF expressed by native NP cells is inactive in nondegenerate samples, and during degeneration NGF is able to signal a response through its receptor TrkA in an autocrine manner. TrkA has also been shown in cultured NP cells following isolation from degenerate IVDs and could be induced by culture in neural differentiation media [51], suggesting maintenance of NGF sensitivity in culture. BDNF mRNA expression levels were significantly higher within the degenerate and infiltrated groups as opposed to the nondegenerate cohort; likewise NGF receptor TrkB was also up regulated in degenerate and infiltrated groups, suggesting increased responsiveness to BDNF within degeneration. Within the current study the majority of degenerate discs displayed TrkB gene expression. Navone and colleagues also demonstrated TrkB protein in cultured NP cells, which was significantly increased by culture in neural differentiation media [51]. The expression of neurotrophic factor receptors by native NP cells raises the question of what effects the neurotrophins play on NP cells. Articular chondrocytes were also shown recently to express the TrkA receptor, and NGF stimulation of a chondrocyte cell line prevented cellular differentiation, suggesting a direct role in chondrocytic cell maturation [52]. Further work is necessary to further elucidate the role of NGF on NP cells and articular chondrocytes.

Peptide containing nerve fibres were first identified within the IVD by Freemont and colleagues [1], who demonstrated SP-expressing nerve fibres within deep tissue of pain-level discs. SP and CGRP are neuropeptides found within dorsal root ganglia neurons and C-fibres of sensory neurons, ultimately involved in the facilitation of pain perception within the peripheral and central nervous system. In addition, CGRP has been shown to induce proliferation of endothelial cells [53], suggesting that expression by NP cells could also induce the angiogenesis seen in disc degeneration. Here, we demonstrate that SP is produced by NP cells and was seen in more degenerate samples than nondegenerate NP samples, with levels increasing in infiltrated discs compared with degenerate discs. Similarly, Richardson and colleagues demonstrated increased SP gene expression in surgical degenerate discs compared with PM discs [50]; these studies suggest that SP is increased during degeneration, which could lead to increased pain sensation within ingrowing nerves.

Following the identification of neurotophic and angiogenic factors within degenerate IVDs, the regulation of these factors by key cytokines that have been shown previously to be upregulated during IVD degeneration was investigated to determine the regulation of neurotrophic and angiogenic factors by potential responsive cells within the disc. Cytokines have been widely studied in IVD degeneration and are known to stimulate the production of catabolic enzymes, consequently leading to the loss of matrix molecules, particularly aggrecan and collagen type II [18,54], which are essential for maintaining the IVD. The early loss of aggrecan is a characteristic feature of IVD degeneration, and the hypothesis is that this loss could in fact lead to the unopposed entry of blood vessels and nerves into the usually avascular and aneural inner AF [43,45,55-57]. IL-1β, IL-6, IL-8 and TNFα are potent inflammatory mediators present within disc degeneration [12,13,15,17,18,35,41,58] and, as shown in this study, cytokines are able to mediate factors involved in innervation, pain transmission and angiogenesis; this confirms regulation of NGF and BDNF seen previously following cytokine treatments of IVD cells [26,30,32,34,59]. This research demonstrates the potential relationships between human NP cells, nerves and blood vessels at the mRNA level and how cytokines may mediate their ingrowth. The regulation of these factors by cytokines also agrees with previous research by Lee and colleagues [34], which showed positive correlations between IL-1β expression in human IVD tissues and that of VEGF, NGF and BDNF protein expression.

The current study identified high levels of SP at mRNA level within human NP cells, which is significantly upregulated by IL-1β stimulation *in vitro*. In contrast, Purmessur and colleagues found SP expression from NP cells was significantly downregulated by 10 ng/ml IL-1β and significantly upregulated by TNFα [30]. To further confirm the upregulation of SP by IL-1β in this study, conditioned medium collected from alginate bead cultures stimulated with IL-1β was investigated for SP protein expression. IL-1β was shown to increase SP levels to 400 pg/ml, suggesting IL-1β is able to actively regulate the release of SP from NP cells.

The production of SP within other cell types has demonstrated dose-dependent increases in the secretion of proinflammatory cytokines via the activation of mitogen-activated protein kinases (p38 and c-jun N terminal kinase), and nuclear factor kappa beta (NFκβ). IL-1β, IL-6, IL-8 and TNFα are known targets of the NFκβ signalling pathway [60,61], which is involved in the production of ADAMTS-4 and ADAMTS-5 [62]. The release of SP from NP cells into the matrix could thus potentially upregulate production of proinflammatory cytokines via activation of the NFκβ pathway. NP cells producing inflammatory cytokines may cause an indirect effect on nerves and blood vessels that are known to express SP receptor neurokinin-1 [63] via the production and upregulation of SP, which could act in an autocrine/paracrine manner within the degenerate IVD, intensifying the targeted destruction of extracellular matrix as well as sensitisation of ingrowing nerves.

Interestingly, neither nerve cells nor endothelial cells stimulated with cytokines displayed regulation of neurotrophic or angiogenic factor expression, which was in stark contrast to the responses seen within stimulated NP cells. The lack of response seen in these cell types could represent the lack of regulation of neurotrophic and angiogenic factors by native blood vessels and of nerve fibres that ingrow into the disc by cytokines. However, the clear regulation of neurotrophic and angiogenic factors by cytokines, particularly IL-1β, suggest these factors play a major role in the neurotrophic and angiogenic factor expression within degenerate discs that contain a rich source of cytokines. This increase in neurotrophic factors by degenerate discs, confirmed by the number of discs containing these factors in degeneration, suggests they play a major role in the regulation of nerve and blood vessel ingrowth in the degenerate disc. To date, few studies have investigated the production of neurotrophic factor expression by SH-SY5Y cells in response to cytokines. Moon and colleagues investigated cytokine stimulation of SH-SY5Y cells co-cultured with AF cells; they demonstrated increased production of NGF in cytokine-treated co-cultures but not in SH-SY5Y cells alone [59], which agrees with the results of this study. Their results suggest either that co-culture with AF cells resulted in co-stimulation of NGF expression by the SH-SY5Y cells or that the increased production of NGF was produced by the AF cells rather than the SH-SY5Y cells [59], as seen in the current study with NP cells. This is the first study to date that has investigated the induction by cytokines of neurotrophic factor expression by endothelial cells. Whilst we showed these cells expressed neurotrophic factors CGRP and VEGF, which confirms the results of previous studies [6,64], they were not regulated by the cytokines investigated within this study. A recent study by Moon and colleagues demonstrated that microvascular endothelial cells cultured in AF conditioned media displayed an increase in NGF protein production, and they suggested IL-8 or VEGF as the secreted factors involved in activation [65]. However, the results from this current study demonstrated that IL-8 had no effect on endothelial cells, suggesting VEGF may be the key player.

## Conclusions

The release of cytokines, particularly IL-1β, during IVD degeneration induced significant increases of NGF at the mRNA level, which is hypothesised to promote neuronal proliferation, migration and survival within the degenerate disc. Not only does IL-1β significantly increase neurotrophic factors but angiogenic growth factor VEGF was also significantly upregulated by IL-1β stimulation, which would potentiate the ingrowth of endothelial cells known to express NGF, which further promotes innervation. Endothelial cell expression of neurotrophic factors was not regulated by cytokine treatment, suggesting that cytokines affect expression of these factors within NP cells more than nerves and blood vessels, leading to the hypothesis that NP cells could facilitate the destruction of the IVD by increasing their expression of NGF and VEGF in response to IL-1β and potentially other cytokines, targeting neurons and endothelial cells that are known to express their receptors. SP that is released into the matrix could potentially upregulate the production of matrix-degrading enzymes and also sensitise nerves, resulting in nociceptive transmission and chronic low back pain. The current study suggests IL-1β as the key regulatory cytokine involved in the upregulation of factors involved in innervation and vascularisation of tissues, whereas IL-6 and TNFα did not have any significant effects on neurotrophic regulation in NP cells in this study. These data are derived from *in vitro* studies and to date the specific concentrations of these cytokines has not been determined *in vivo*, however, so it must be considered that other cytokines which could be expressed at high levels in the disc may also play a role. The majority of the cytokine-mediated responses were in disc cells, whilst nerve and endothelial cells show minimal effects. NP cells could potentially regulate endothelial cell ingrowth into the degenerate IVD via the activation of various pathways involved in the increase of catabolic factors and upregulation of peptides known to sensitise nerves, ultimately leading to chronic low back pain within IVD degeneration.

## Abbreviations

ADAMTs: a disintegrin and metalloproteinase with thrombospondin motifs
AF: annulus fibrosus
BDNF: brain-derived neurotrophic factor
CGRP: calcitonin gene-related peptide
HDMEC: human dermal microvascular endothelial cells
IL: interleukin
IVD: intervertebral disc
MMP: matrix metalloproteinase
NFκB: nuclear factor kappa beta
NGF: nerve growth factor
NP: nucleus pulposus
NT3: neurotrophin 3
qRT-PCR: quantitative real-time polymerase chain reaction
SP: substance P
TNFα: tumour necrosis factor alpha
Trk: tropomyosin kinase
VEGF: vascular endothelial growth factor

## References

[1] Freemont AJ, Peacock TE, Goupille P, Hoyland JA, O'Brien J, Jayson MI (1997). Nerve ingrowth into diseased intervertebral disc in chronic back pain. Lancet.

[2] Coppes MH, Marani E, Thomeer RT, Groen GJ (1997). Innervation of ‘painful’ lumbar discs. Spine.

[3] Palmgren T, Gronblad M, Virri J, Seitsalo S, Ruuskanen M, Karaharju E (1996). Immunohistochemical demonstration of sensory and autonomic nerve terminals in herniated lumbar disc tissue. Spine (Phila Pa 1976).

[4] Luoma K, Riihimaki H, Luukkonen R, Raininko R, Viikari-Juntura E, Lamminen A (2000). Low back pain in relation to lumbar disc degeneration. Spine (Phila Pa 1976).

[5] Maniadakis N, Gray A (2000). The economic burden of back pain in the UK. Pain.

[6] Freemont AJ, Watkins A, Le Maitre C, Baird P, Jeziorska M, Knight MT, Ross ER, O'Brien JP, Hoyland JA (2002). Nerve growth factor expression and innervation of the painful intervertebral disc. J Pathol.

[7] Ohtori S, Inoue G, Miyagi M, Takahashi K: **Pathomechanisms of discogenic low back pain in humans and animal models.** *Spine J* 2014, doi:10.1016/j.spinee.2013.07.490. 10.1016/j.spinee.2013.07.490 24657737

[8] Risbud MV, Shapiro IM (2014). Role of cytokines in intervertebral disc degeneration: pain and disc content. Nat Rev Rheumatol.

[9] Le Maitre CL, Pockert A, Buttle DJ, Freemont AJ, Hoyland JA (2007). Matrix synthesis and degradation in human intervertebral disc degeneration. Biochem Soc Trans.

[10] Le Maitre CL, Freemont AJ, Hoyland JA (2007). Accelerated cellular senescence in degenerate intervertebral discs: a possible role in the pathogenesis of intervertebral disc degeneration. Arthritis Res Ther.

[11] Ahn CH, Cho YW, Ahn MW, Jang SH, Sohn YK, Kim HS (2002). mRNA expression of cytokines and chemokines in herniated lumbar intervertebral discs. Spine.

[12] Purmessur D, Walter BA, Roughley PJ, Laudier DM, Hecht AC, Iatridis J (2013). A role for TNFalpha in intervertebral disc degeneration: a non-recoverable catabolic shift. Biochem Biophys Res Commun.

[13] Andrade P, Hoogland G, Garcia MA, Steinbusch HW, Daemen MA, Visser-Vandewalle V (2013). Elevated IL-1beta and IL-6 levels in lumbar herniated discs in patients with sciatic pain. Eur Spine J.

[14] Burke JG, Watson RW, McCormack D, Dowling FE, Walsh MG, Fitzpatrick JM (2002). Intervertebral discs which cause low back pain secrete high levels of proinflammatory mediators. J Bone Joint Surg (Br).

[15] Weiler C, Nerlich AG, Bachmeier BE, Boos N (2005). Expression and distribution of tumor necrosis factor alpha in human lumbar intervertebral discs: a study in surgical specimen and autopsy controls. Spine (Phila Pa 1976).

[16] Takahashi H, Suguro T, Okazima Y, Motegi M, Okada Y, Kakiuchi T (1996). Inflammatory cytokines in the herniated disc of the lumbar spine. Spine (Phila Pa 1976).

[17] Le Maitre CL, Hoyland JA, Freemont AJ (2007). Catabolic cytokine expression in degenerate and herniated human intervertebral discs: IL-1beta and TNFalpha expression profile. Arthritis Res Ther.

[18] Le Maitre CL, Freemont AJ, Hoyland JA (2005). The role of interleukin-1 in the pathogenesis of human intervertebral disc degeneration. Arthritis Res Ther.

[19] Le Maitre CL, Freemont AJ, Hoyland JA (2004). Localization of degradative enzymes and their inhibitors in the degenerate human intervertebral disc. J Pathol.

[20] Pockert AJ, Richardson SM, Le Maitre CL, Lyon M, Deakin JA, Buttle DJ, Freemont AJ, Hoyland JA (2009). Modified expression of the ADAMTS enzymes and tissue inhibitor of metalloproteinases 3 during human intervertebral disc degeneration. Arthritis Rheum.

[21] Hoyland JA, Le Maitre C, Freemont AJ (2008). Investigation of the role of IL-1 and TNF in matrix degradation in the intervertebral disc. Rheumatology (Oxford).

[22] Roberts S, Caterson B, Menage J, Evans EH, Jaffray DC, Eisenstein SM (2000). Matrix metalloproteinases and aggrecanase: their role in disorders of the human intervertebral disc. Spine (Phila Pa 1976).

[23] Zhao CQ, Zhang YH, Jiang SD, Li H, Jiang LS, Dai LY (2011). ADAMTS-5 and intervertebral disc degeneration: the results of tissue immunohistochemistry and in vitro cell culture. J Orthop Res.

[24] Patel KP, Sandy JD, Akeda K, Miyamoto K, Chujo T, An HS, Masuda K (2007). Aggrecanases and aggrecanase-generated fragments in the human intervertebral disc at early and advanced stages of disc degeneration. Spine (Phila Pa 1976).

[25] Deyo RA, Weinstein JN (2001). Low back pain. N Engl J Med.

[26] Gruber HE, Hoelscher GL, Bethea S, Hanley EN (2012). Interleukin 1-beta upregulates brain-derived neurotrophic factor, neurotrophin 3 and neuropilin 2 gene expression and NGF production in annulus cells. Biotech Histochem.

[27] Gruber HE, Hoelscher GL, Ingram JA, Hanley EN (2012). Genome-wide analysis of pain-, nerve- and neurotrophin-related gene expression in the degenerating human annulus. Mol Pain.

[28] Gruber HE, Ingram JA, Hoelscher G, Zinchenko N, Norton HJ, Hanley EN (2008). Brain-derived neurotrophic factor and its receptor in the human and the sand rat intervertebral disc. Arthritis Res Ther.

[29] Richardson SM, Purmessur D, Baird P, Probyn B, Freemont AJ, Hoyland JA (2012). Degenerate human nucleus pulposus cells promote neurite outgrowth in neural cells. PLoS One.

[30] Purmessur D, Freemont AJ, Hoyland JA (2008). Expression and regulation of neurotrophins in the nondegenerate and degenerate human intervertebral disc. Arthritis Res Ther.

[31] Woolf CJ, Safieh-Garabedian B, Ma QP, Crilly P, Winter J (1994). Nerve growth factor contributes to the generation of inflammatory sensory hypersensitivity. Neuroscience.

[32] Abe Y, Akeda K, An HS, Aoki Y, Pichika R, Muehleman C, Kimura T, Masuda K (2007). Proinflammatory cytokines stimulate the expression of nerve growth factor by human intervertebral disc cells. Spine (Phila Pa 1976).

[33] Johnson WE, Patterson AM, Eisenstein SM, Roberts S (2007). The presence of pleiotrophin in the human intervertebral disc is associated with increased vascularization: an immunohistologic study. Spine (Phila Pa 1976).

[34] Lee JM, Song JY, Baek M, Jung HY, Kang H, Han IB, Kwon YD, Shin DE (2011). Interleukin-1beta induces angiogenesis and innervation in human intervertebral disc degeneration. J Orthop Res.

[35] Phillips KL, Chiverton N, Michael AL, Cole AA, Breakwell LM, Haddock G, Bunning RA, Cross AK, Le Maitre CL (2013). The cytokine and chemokine expression profile of nucleus pulposus cells: implications for degeneration and regeneration of the intervertebral disc. Arthritis Res Ther.

[36] Roberts S, Evans H, Trivedi J, Menage J (2006). Histology and pathology of the human intervertebral disc. J Bone Joint Surg Am.

[37] Wang H, Tian Y, Wang J, Phillips KL, Binch AL, Dunn S, Cross A, Chiverton N, Zheng Z, Shapiro IM, Le Maitre CL, Risbud MV (2013). Inflammatory cytokines induce NOTCH signaling in nucleus pulposus cells: implications in intervertebral disc degeneration. J Biol Chem.

[38] Wang JY, Baer AE, Kraus VB, Setton LA (2001). Intervertebral disc cells exhibit differences in gene expression in alginate and monolayer culture. Spine (Phila Pa 1976).

[39] Livak KJ, Schmittgen TD (2001). Analysis of relative gene expression data using real-time quantitative PCR and the 2(−Delta Delta C(T)) method. Methods.

[40] Bachmeier BE, Nerlich AG, Weiler C, Paesold G, Jochum M, Boos N (2007). Analysis of tissue distribution of TNF-alpha, TNF-alpha-receptors, and the activating TNF-alpha-converting enzyme suggests activation of the TNF-alpha system in the aging intervertebral disc. Ann N Y Acad Sci.

[41] Phillips KL, Jordan-Mahy N, Nicklin MJ, Le Maitre CL (2013). Interleukin-1 receptor antagonist deficient mice provide insights into pathogenesis of human intervertebral disc degeneration. Ann Rheum Dis.

[42] Garcia-Cosamalon J, del Valle ME, Calavia MG, Garcia-Suarez O, Lopez-Muniz A, Otero J, Vega JA (2010). Intervertebral disc, sensory nerves and neurotrophins: who is who in discogenic pain?. J Anat.

[43] Johnson WE, Sivan S, Wright KT, Eisenstein SM, Maroudas A, Roberts S (2006). Human intervertebral disc cells promote nerve growth over substrata of human intervertebral disc aggrecan. Spine (Phila Pa 1976).

[44] Peng B, Wu W, Hou S, Li P, Zhang C, Yang Y (2005). The pathogenesis of discogenic low back pain. J Bone Joint Surg (Br).

[45] Melrose J, Roberts S, Smith S, Menage J, Ghosh P (2002). Increased nerve and blood vessel ingrowth associated with proteoglycan depletion in an ovine anular lesion model of experimental disc degeneration. Spine (Phila Pa 1976).

[46] Ohtori S, Takahashi K, Chiba T, Yamagata M, Sameda H, Moriya H (2002). Substance P and calcitonin gene-related peptide immunoreactive sensory DRG neurons innervating the lumbar intervertebral discs in rats. Ann Anat.

[47] Ashton IK, Roberts S, Jaffray DC, Polak JM, Eisenstein SM (1994). Neuropeptides in the human intervertebral disc. J Orthop Res.

[48] Stefanakis M, Al-Abbasi M, Harding I, Pollintine P, Dolan P, Tarlton J, Adams MA (2012). Annulus fissures are mechanically and chemically conducive to the ingrowth of nerves and blood vessels. Spine (Phila Pa 1976).

[49] Krock E, Rosenzweig DH, Chabot-Dore AJ, Jarzem P, Weber MH, Ouellet JA, Stone LS, Haglund L (2014). Painful, degenerating intervertebral discs up-regulate neurite sprouting and CGRP through nociceptive factors. J Cell Mol Med.

[50] Richardson SM, Doyle P, Minogue BM, Gnanalingham K, Hoyland JA (2009). Increased expression of matrix metalloproteinase-10, nerve growth factor and substance P in the painful degenerate intervertebral disc. Arthritis Res Ther.

[51] Navone SE, Marfia G, Canzi L, Ciusani E, Canazza A, Visintini S, Campanella R, Parati EA (2012). Expression of neural and neurotrophic markers in nucleus pulposus cells isolated from degenerated intervertebral disc. J Orthop Res.

[52] Huang H, Shank G, Ma L, Tallents RH, Kyrkanides S (2013). Nerve growth factor induced after temporomandibular joint inflammation decelerates chondrocyte differentiation. Oral Dis.

[53] Tuo Y, Guo X, Zhang X, Wang Z, Zhou J, Xia L, Zhang Y, Wen J, Jin D (2013). The biological effects and mechanisms of calcitonin gene-related peptide on human endothelial cell. J Recept Signal Transduct Res.

[54] Sive JI, Baird P, Jeziorsk M, Watkins A, Hoyland JA, Freemont AJ (2002). Expression of chondrocyte markers by cells of normal and degenerate intervertebral discs. Mol Pathol.

[55] Melrose J, Smith SM, Appleyard RC, Little CB (2008). Aggrecan, versican and type VI collagen are components of annular translamellar crossbridges in the intervertebral disc. Eur Spine J.

[56] Neidlinger-Wilke C, Liedert A, Wuertz K, Buser Z, Rinkler C, Kafer W, Ignatius A, Claes L, Roberts S, Johnson WE (2009). Mechanical stimulation alters pleiotrophin and aggrecan expression by human intervertebral disc cells and influences their capacity to stimulate endothelial migration. Spine (Phila Pa 1976).

[57] Johnson WE, Caterson B, Eisenstein SM, Roberts S (2005). Human intervertebral disc aggrecan inhibits endothelial cell adhesion and cell migration in vitro. Spine (Phila Pa 1976).

[58] Le Maitre CL, Hoyland JA, Freemont AJ (2007). Interleukin-1 receptor antagonist delivered directly and by gene therapy inhibits matrix degradation in the intact degenerate human intervertebral disc: an in situ zymographic and gene therapy study. Arthritis Res Ther.

[59] Moon HJ, Kim JH, Lee HS, Chotai S, Kang JD, Suh JK, Park YK (2012). Annulus fibrosus cells interact with neuron-like cells to modulate production of growth factors and cytokines in symptomatic disc degeneration. Spine (Phila Pa 1976).

[60] Azzolina A, Guarneri P, Lampiasi N (2002). Involvement of p38 and JNK MAPKs pathways in substance P-induced production of TNF-alpha by peritoneal mast cells. Cytokine.

[61] Ulrich JA, Liebenberg EC, Thuillier DU, Lotz JC (2007). ISSLS prize winner: repeated disc injury causes persistent inflammation. Spine (Phila Pa 1976).

[62] Tian Y, Yuan W, Fujita N, Wang J, Wang H, Shapiro IM, Risbud MV (2013). Inflammatory cytokines associated with degenerative disc disease control aggrecanase-1 (ADAMTS-4) expression in nucleus pulposus cells through MAPK and NF-kappaB. Am J Pathol.

[63] Ashton IK, Walsh DA, Polak JM, Eisenstein SM (1994). Substance P in intervertebral discs, binding sites on vascular endothelium of the human annulus fibrosus. Acta Orthop Scand.

[64] Fang L, Chen MF, Xiao ZL, Liu Y, Yu GL, Chen XB, Xie XM (2011). Calcitonin gene-related peptide released from endothelial progenitor cells inhibits the proliferation of rat vascular smooth muscle cells induced by angiotensin II. Mol Cell Biochem.

[65] Moon HJ, Yurube T, Lozito TP, Pohl P, Hartman RA, Sowa GA, Kang JD, Vo NV (2014). Effects of secreted factors in culture medium of annulus fibrosus cells on microvascular endothelial cells: elucidating the possible pathomechanisms of matrix degradation and nerve in-growth in disc degeneration. Osteoarthritis Cartilage.

